# Outcome following Resection of Biliary Cystadenoma: A Single Centre Experience and Literature Review

**DOI:** 10.1155/2015/382315

**Published:** 2015-12-29

**Authors:** M. Pitchaimuthu, G. Aidoo-Micah, C. Coldham, R. Sutcliffe, J. K. Roberts, P. Muiesan, J. Isaac, D. Mirza, R. Marudanayagam

**Affiliations:** Liver and HPB Unit, Queen Elizabeth Hospital, University Hospitals Birmingham NHS Foundation Trust, Birmingham B15 2TH, UK

## Abstract

*Background*. Biliary cystadenomas (BCAs) are rare, benign, potentially malignant cystic lesions of the liver, accounting for less than 5% of cystic liver tumours. We report the outcome following resection of biliary cystadenoma from a single tertiary centre.* Methods*. Data of patients who had resection of BCA between January 1993 and July 2014 were obtained from liver surgical database. Patient demographics, clinicopathological characteristics, operative data, and postoperative outcome were analysed.* Results*. 29 patients had surgery for BCA. Male : female ratio was 1 : 28. Clinical presentation was abdominal pain (74%), jaundice (20%), abdominal mass (14%), and deranged liver function tests (3%). Cyst characteristics included septations (48%), wall thickening (31%), wall irregularity (38%), papillary projections (10%), and mural nodule (3%). Surgical procedures included atypical liver resection (52%), left hemihepatectomy (34%), right hemihepatectomy (10%), and left lateral segmentectomy (3%). Median length of stay was 7 (IQ 6.5–8.5) days. Two patients developed postoperative bile leak. No patients had malignancy on final histology. Median follow-up was 13 (IQ 6.5–15.7) years. One patient developed delayed biliary stricture and one died of cholangiocarcinoma 11 years later.* Conclusion.* Biliary cystadenomas can be resected safely with significantly low morbidity. Malignant transformation and recurrence are rare. Complete surgical resection provides a cure.

## 1. Introduction

Biliary cystadenomas (BCAs) are rare, benign, but potentially malignant unilocular or multilocular cystic lesions of the biliary system occurring most commonly in women [1–5]. It commonly arises from the liver, but BCAs of extra hepatic biliary tract and gall bladder have been reported [2, 6–9]. BCAs account for approximately 5% of all hepatic cystic lesions [10].

First publication on BCA was in 1887 and the first resection was performed in 1892 [11]. With the widespread availability of modern imaging techniques and developments in safe liver surgery, the number of reported cases increased in 1990s [12]. Diagnosis of BCA was complicated by the presence of wide variety of cystic lesions in the liver, including simple cysts, parasitic cysts, degenerated metastatic tumours, mucin-producing metastatic tumours, congenital cystic dilatations, cystic hemangioma, lymphangioma, hepatic foregut cysts, mesenchymal hamartoma, and teratoma [5, 13–15].

Due to its premalignant nature and lack of reliability of imaging and preoperative aspiration cytology in differentiating BCA from cystadenocarcinoma, all lesions suspected of BCA should be surgically removed with negative margin [14–21]. We aim to describe and analyse the outcome following resection of BCA from a single tertiary centre.

## 2. Methods

A retrospective analysis of all patients who had resection of BCA was carried out from a prospectively maintained liver surgical and pathological database over a period of 21 years (January 1993 to July 2014). Patients' demographics, clinical presentation, preoperative imaging, operative data and postoperative outcome were analysed. Cystic lesions of the liver were evaluated with ultrasound scan, CT scan, and MRI liver (in selected cases). Cyst characteristics were analysed with respect to number of cysts (single/multiple), locularity (unilocular or multilocular), presence of cyst wall thickening, irregularity, papillary projections, and mural nodule. Cystic lesions showing wall thickening, irregularity, internal septations, presence of nodules, papillary projections, and communication with biliary system were offered surgical resection. Nonanatomical/segmental resection was performed if feasible, but large BCAs and lesions confined to few segments were treated with formal right or left hepatectomy or left lateral segmentectomy. Postoperative complications were recorded according to Dindo et al. classification [22]. Histopathological information regarding completeness of excision, resection margin, and evidence of dysplasia/malignancy were collated. Patients who had partial excision of cyst wall for suspected simple cyst and subsequently found to have BCA were subjected to formal resection. All patients were followed up in the clinic within 6 weeks of surgery and were reviewed in the clinic if necessary thereafter. Patients latest follow-up details were collected from the hospital based computer system and where there was no information available, patient's general practitioners were contacted regarding patients current clinical status.

## 3. Statistical Analysis

Continuous variables were presented as median with interquartile range and the categorical variables were presented in percentages. Survival was calculated using Kaplan-Meier method. SPSS version 22.0 (SPSS, Chicago, IL) was used to perform statistical analysis.

## 4. Results

187 patients with cystic liver lesions underwent surgery during the study period. Of these, 29 patients were found to have BCA. Median age was 62 (IQ 48–74) years and all were women except one. The main clinical presentation was abdominal pain (74%) followed by jaundice (20%), abdominal mass (14%), and deranged liver function tests (LFTs) (3%). Patients who presented with deranged LFTs mainly had elevated transaminases with normal bilirubin. One patient was incidentally found to have a cystic lesion in the liver on ultrasound scan performed for irritable bowel syndrome like symptoms.

On preoperative imaging, single cyst was seen in majority of the patients (76%). Approximately half of the cysts were unilocular (*n* = 15). Cysts were centrally located in 48% of the cases. Cyst characteristics were as follows: wall thickening (31%), wall irregularity (38%), papillary projections (10%), and mural nodule (3%). Twenty-six (89%) patients were suspected of having BCA on preoperative imaging while the remaining 3 patients were suspected to have simple liver cysts. None of the patients had preoperative cyst fluid cytology, as we believe that negative result will not preclude surgery.

Surgical procedures performed were as follows: nonanatomical liver resection (52%), left hemihepatectomy (34%), right hemihepatectomy (10%), and left lateral segmentectomy (3%). Three patients had formal reresection following histological confirmation of BCA after deroofing of suspected simple liver cysts. Median length of stay was 7 (IQ 6.5–8.5) days. Two patients developed postoperative bile leak. One patient had a low volume bile leak, which was treated conservatively. The other patient developed bile leak following deroofing of suspected simple liver cyst and required an endoscopic biliary stenting to control the high volume bile leak. This patient subsequently had a formal resection following histological confirmation of BCA. Two and three patients each developed wound and chest infection, all of which were treated with antibiotics. There was no perioperative mortality.

On pathological examination, the median size of the cyst was 12 (IQ 8.5–18) cm. On histology, 22 patients had typical ovarian/mesenchymal stroma diagnostic of BCA and 6 patients had no mesenchymal stroma but were positively stained for mucin. Three patients had features of papillary tufting (IPNB) with 2 of them also having the presence of ovarian stroma. Two patients showed calcification in addition to ovarian stroma. None of these patients had evidence of malignancy on final histology.

Median follow-up was 13 (IQ 6.5–15.7) years. One patient developed delayed biliary stricture three years after surgery. This was initially treated with percutaneous transhepatic cholangiogram (PTC) guided dilatation but eventually required Roux-en-Y hepaticojejunostomy, 7 years from the primary surgery. None of our patients developed recurrence of BCA during follow-up. One patient died 11 years later due to development of inoperable cholangiocarcinoma at a different site.

## 5. Discussion

Biliary cystadenomas (BCAs) are potentially malignant, rare benign cystic lesions of the biliary system of unknown aetiology [10, 23]. The incidence of BCA is from one in 20,000 to 100,000 people, while the occurrence of cystadenocarcinoma is approximately one in 10 million patients [17]. Even though the previously published literature quoted the incidence of BCAs to be around 5% of cystic hepatic tumours, the true incidence is still unclear [12, 15, 24]. The controversy is due to lack of established criteria for preoperative diagnosis of unilocular BCAs [6, 12, 14, 16]. Due to its potential for malignant transformation and tendency to recur, there is a great concern in treating this tumour with techniques other than formal resection [3–5].

Aetiology of BCA is unclear and both congenital and acquired origins have been proposed. Presence of hamartomatous structures supports the theory of congenital origin [25]. Cruickshank et al. proposed a theory of acquired lesion and reported the development of BCAs as a reactive process to focal injury [3, 26]. It was also suggested that this might have arisen from ectopic embryonal tissue destined to form gall bladder or from ectopic embryonic rest of primitive foregut sequestered within the liver [3, 24, 27, 28]. Presence of endocrine cells in BCAs and cystadenocarcinomas also suggest the origin from peribiliary glands [29].

Biliary cystadenomas are composed of communicating variable sized locules usually containing clear fluid. The locules are lined by simple, predominantly columnar epithelium resembling biliary epithelium with cytoplasmic mucin [23]. Edmondson initially defined BCAs in 1958 as multilocular lesions with ovarian-like stroma, but subsequently BCAs without ovarian stroma were reported [5]. Due to this uncertainty WHO, which initially classified these lesions as biliary cystadenoma/adenocarcinoma in 2000, redefined these lesions as either mucinous cystic neoplasm (MCN) or intraductal papillary neoplasm of bile duct (IPN-B) depending on the presence of ovarian stroma and papillary growth within the bile duct, respectively [30–32]. WHO further classified these IPNBs into IPN with low- or intermediate-grade intraepithelial neoplasia, IPN with high-grade intraepithelial neoplasia, and IPN with an associated invasive carcinoma [33]. The majority of biliary cystadenomas do not communicate with the bile ducts, but luminal communication may be occasionally observed [34].

The epithelium may show varying degrees of dysplasia. High-grade dysplasia or invasive carcinoma suggest transformation to cystadenocarcinoma [23]. Devaney et al. proposed three subsets of cystadenocarcinoma based on the pathology material submitted to their institutional laboratories for primary diagnosis or consultation: (1) cystadenocarcinoma originating from a benign cystadenoma with ovarian-like stroma occurs exclusively in women; (2) de novo cystadenocarcinoma occurs almost only in men; and (3) cystadenocarcinoma occurs in women but does not contain an ovarian-like stroma [5].

The development of cystadenocarcinomas is unclear, whether they are de novo cancers or develop from preexisting BCAs. However, it is generally believed that they arise from BCAs as many cystadenocarcinomas contain areas of BCAs in the same sample [3, 24, 28, 35]. A malignant transformation rate of up to 30% has been reported, and biliary cystadenocarcinoma accounts for 0.41% of malignant hepatic epithelial tumours [15, 16, 18, 19, 36, 37]. Elements of BCA were present in 1/3 of biliary cystadenocarcinomas [5, 24]. Wheeler and Edmondson reported malignant change in 23% of the resected BCAs [24]. In our study none of the patients had malignant change in the final histology of resected BCAs. But there were no case-control or cohort studies to define the exact risk of malignant transformation of untreated biliary cystadenoma to cystadenocarcinoma. In the present study also it is unclear, as our patient developed cholangiocarcinoma rather than cystadenocarcinoma many years after the resection of BCA.

Majority of the BCAs present with vague abdominal complaints secondary to extrinsic compression of stomach, duodenum, or biliary tree [5, 24, 38]. In our series, about three quarters of the patients presented with vague abdominal pain. But BCAs have also been identified in asymptomatic patients during imaging or surgical exploration for unrelated clinical conditions [39].

Even with improved imaging techniques, it is still challenging to differentiate BCAs from other cystic lesions of the liver. Differential diagnosis for BCAs includes simple liver cysts, parasitic cysts, haematomas or posttraumatic cysts, liver abscesses, congenital cysts, polycystic disease, Caroli's disease, and neoplastic lesions such as biliary cystadenocarcinoma, undifferentiated embryonal sarcoma, cystic metastasis, metastatic pancreatic or ovarian cystadenocarcinoma, biliary papilloma (IPNB), cystic primary hepatocellular carcinoma, cystic cholangiocarcinoma, and hepatobiliary mesenchymal tumours [2, 32, 40, 41].

**Figure 1:**
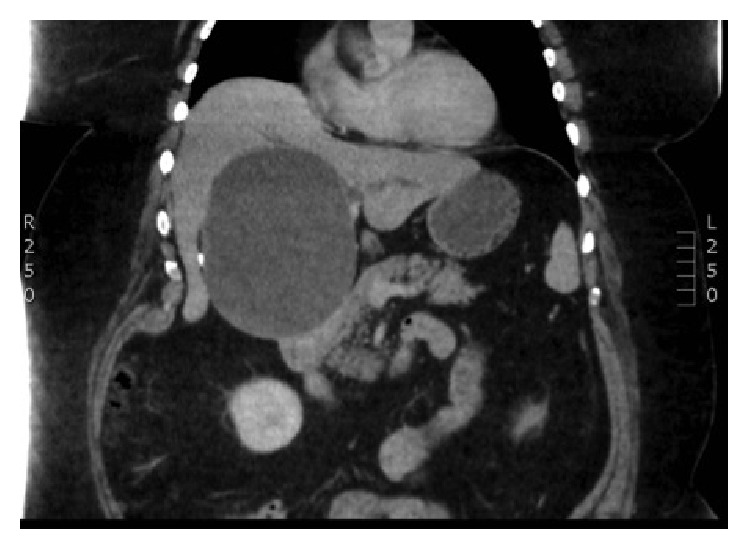
CT scan showing unilocular biliary cystadenoma without any septations but with calcification in the wall.

**Figure 2:**
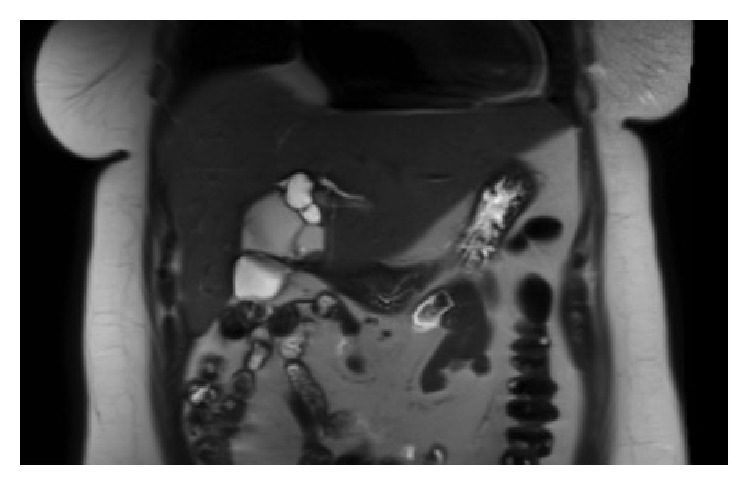
MRI picture showing septated biliary cystadenoma with nodule.

Ultrasound scan, CT (Figure 1), MRI (Figure 2), MRCP, ERCP, intraoperative cholangiography, and choledochoscopy all have been described in the literature for the preoperative work-up of BCA [42–46]. Preoperative radiological diagnostic accuracy may be as low as 30%, so a high index of suspicion is required in the diagnosis of BCA [47].

Although radiologic features such as papillary projections, internal septations, wall thickness, irregularities, and mural nodules suggest the possibility of a BCA, all of these except papillary projections may be observed in simple cysts as well albeit at a lower frequency [48, 49].

Preoperative cyst fluid aspiration for diagnosis has been advocated in the published literature. Cyst fluid CA19-9 and CEA levels can be helpful to enhance the accuracy of diagnosis of BCAs and cystadenocarcinomas from other cystic lesions [10, 17, 50]. However, it is not accurate in differentiating BCAs from cystadenocarcinoma, as inadequate sampling may miss the microscopic foci of carcinoma in a cystadenoma [50–52]. Preoperative differentiation of BCAs from cystadenocarcinoma is extremely difficult and can only be done after pathological examination [1, 3, 24, 28, 39, 41]. FNA and needle biopsy may risk dissemination of tumour cells and it is not generally recommended, especially when surgery is planned [10, 52].

Improper preoperative diagnosis can lead to incorrect and unnecessary procedures such as percutaneous aspiration, ethanol injection, deroofing, and omentoplasty [20]. In our series, 3 patients were diagnosed as simple cysts on imaging but on histological examination were found to be BCAs. These patients had second surgical intervention to remove the lesion with clear margins.

Lewis et al. and Madariaga et al. recommended surgical resection as the treatment of choice for all multiloculated hepatic cystic lesions [16, 53]. If BCA is suspected on imaging, surgery is indicated even if the patient is asymptomatic [1, 24, 28, 39, 52, 54]. The extent of resection remains to be determined, as partial resection with occasional ablation of the residual cyst using electrocautery or argon beam coagulation and/or omentopexy, lobectomy, wedge resection, and enucleation have all been reported [16, 17, 28, 29, 55–57]. Pinson et al. have reported cyst enucleation without late recurrence and mortality [57]. This procedure is a valid alternative where resection is difficult or is likely to be associated with morbidity [10, 14]. In our series, majority of the patients had nonanatomical liver resections. Patients with large BCAs and lesions confined to few segments were treated with formal hepatectomy.

Other limited procedures like aspiration, fenestration, internal drainage, sclerotherapy, and partial resection are associated with extremely high recurrence ranging from 90 to 100%, compared to 0 to 10% with formal resection [4, 14, 16, 20, 52, 55, 58–61]. In our series, all of our patients had formal resection with no recurrence after a median follow-up of 13 (IQ 6.5–15.7) years. Due to its premalignant nature, low radiological diagnostic accuracy, and high recurrence with improper treatment, formal liver resection is recommended.

The prognosis of patients with biliary cystadenoma is good if total excision of the lesion is performed [3, 5, 14, 20, 24, 52]. In the present study the 10-year survival was 90%. Only one patient died of late development of cholangiocarcinoma and 2 other patients died of other causes after 8 and 9 years following resection, respectively.

We conclude that biliary cystadenomas are rare and can be resected safely with significantly low morbidity. Malignant change and recurrence are rare. Complete surgical resection provides a cure.

## Abbreviations

BCA: Biliary cystadenoma
MCN: Mucinous cystic neoplasm
IPNB: Intraductal papillary neoplasm of bile duct.
